# Targeting cyclin-dependent kinase 1 (CDK1) but not CDK4/6 or CDK2 is selectively lethal to MYC-dependent human breast cancer cells

**DOI:** 10.1186/1471-2407-14-32

**Published:** 2014-01-20

**Authors:** Jian Kang, C Marcelo Sergio, Robert L Sutherland, Elizabeth A Musgrove

**Affiliations:** 1 The Kinghorn Cancer Centre and Cancer Research Division, Garvan Institute of Medical Research, 370 Victoria Street, Darlinghurst, Sydney, NSW, Australia; 2 St Vincent’s Clinical School, Faculty of Medicine, University of New South Wales, St Vincent’s Hospital, Sydney, NSW, Australia; 3 Wolfson Wohl Cancer Research Centre, University of Glasgow, Garscube Estate, Switchback Road, Bearsden, Glasgow G61 1BD, Scotland, UK

**Keywords:** MYC, Cyclin-dependent kinase, Synthetic lethality, Breast cancer

## Abstract

**Background:**

Although MYC is an attractive therapeutic target for breast cancer treatment, it has proven challenging to inhibit MYC directly, and clinically effective pharmaceutical agents targeting MYC are not yet available. An alternative approach is to identify genes that are synthetically lethal in MYC-dependent cancer. Recent studies have identified several cell cycle kinases as MYC synthetic-lethal genes. We therefore investigated the therapeutic potential of specific cyclin-dependent kinase (CDK) inhibition in MYC-driven breast cancer.

**Methods:**

Using small interfering RNA (siRNA), MYC expression was depleted in 26 human breast cancer cell lines and cell proliferation evaluated by BrdU incorporation. MYC-dependent and MYC-independent cell lines were classified based on their sensitivity to siRNA-mediated MYC knockdown. We then inhibited CDKs including CDK4/6, CDK2 and CDK1 individually using either RNAi or small molecule inhibitors, and compared sensitivity to CDK inhibition with MYC dependence in breast cancer cells.

**Results:**

Breast cancer cells displayed a wide range of sensitivity to siRNA-mediated MYC knockdown. The sensitivity was correlated with MYC protein expression and MYC phosphorylation level. Sensitivity to siRNA-mediated MYC knockdown did not parallel sensitivity to the CDK4/6 inhibitor PD0332991; instead MYC-independent cell lines were generally sensitive to PD0332991. Cell cycle arrest induced by MYC knockdown was accompanied by a decrease in CDK2 activity, but inactivation of CDK2 did not selectively affect the viability of MYC-dependent breast cancer cells. In contrast, CDK1 inactivation significantly induced apoptosis and reduced viability of MYC-dependent cells but not MYC- independent cells. This selective induction of apoptosis by CDK1 inhibitors was associated with up-regulation of the pro-apoptotic molecule BIM and was p53-independent.

**Conclusions:**

Overall, these results suggest that further investigation of CDK1 inhibition as a potential therapy for MYC-dependent breast cancer is warranted.

## Background

The *MYC* oncogene is one of the most commonly amplified oncogenes in human breast cancer and contributes to its formation and development [1-3]. *MYC* gene amplification has been found in approximately 15% of breast tumours, while more than 40% of breast cancers over-express MYC protein, indicating that gene amplification is not the only cause of MYC over-expression [4,5]. MYC over-expression results in a number of cellular changes, including transcriptional amplification [6,7] and increased protein biosynthesis [8]. MYC-stimulated cell cycle progression has also been well studied. Cyclin-dependent kinases (CDKs), including three interphase CDKs (CDK2, CDK4 and CDK6) and a mitotic CDK (CDK1), are critical regulators of cell cycle progression in mammalian cells [9]. Increased cyclin E-CDK2 activity appears to be a principal mechanism contributing to MYC-induced G_1_-S phase transition in breast cancer cells [10,11], possibly through suppression of the CDK inhibitor p21 [12,13] and induction of the CDK phosphatase CDC25A [14]. Although cyclin D1 and CDK4 are putative MYC target genes, and required for MYC-mediated transformation in keratinocytes [15,16], the proliferative effect of MYC in breast cancer cells appears to be independent of cyclin D1/CDK4 activation as evidenced by the absence of cyclin D1 up-regulation and CDK4 activation upon MYC induction [11].

The key role of MYC activation in the pathogenesis of breast cancer and the high incidence of MYC deregulation make MYC an attractive therapeutic target in breast cancer. However, transcription factors such as MYC are challenging to target directly and clinically-effective pharmaceutical agents targeting MYC are not yet available [17,18]. Nevertheless, cancer cells develop dependence on other genes and pathways in order to overcome anti-tumorigenic effects, such as apoptosis and senescence, that result from activation of MYC. These dependencies may provide novel therapeutic options for targeting MYC addiction. Consequently, an alternative approach which has recently received great attention is to identify genes that are synthetically lethal in MYC-dependent cancers. Genome-wide RNAi screens for synthetic lethality in MYC over-expressing cells highlight the potential of targeting cell cycle kinases for MYC-dependent cancers [19,20]. Other studies using a candidate approach also identified several cell cycle kinases as MYC-synthetic lethal genes in different types of cancer, including CDK2 [21], CDK1 [22] and aurora-B kinase [23]. Since cellular context and tissue type affect the biological functions of MYC [24] and thus presumably affect these synthetic lethal interactions, we investigated the therapeutic potential of specific CDK inhibition in MYC-driven breast cancer.

Aberrant CDK activation induces unscheduled proliferation and leads to genomic and chromosomal instability in cancer cells [25]. Consequently, CDK inhibition has been considered as a potential therapeutic strategy for cancer treatment, and a series of CDK inhibitors have been developed. Disappointingly, CDK inhibitors have yet to demonstrate significant clinical advantages as sole agents [26]. Accumulating evidence suggests that tumour cells have a selective dependence on specific CDKs, therefore, identification of specific genetic contexts in which tumour cells are the most likely to be responsive to CDK inhibitors, is required to improve effectiveness of CDK inhibitors in clinical trial [25].

In this study we used an RNAi approach to identify MYC-dependent breast cancer cell lines and then inhibited CDKs including CDK4/6, CDK2 and CDK1 individually by either RNAi or small molecule inhibitors in both MYC-dependent and MYC-independent cells. We found that targeting CDK1 rather than CDK4/6 or CDK2 selectively reduced the viability of MYC- dependent breast cancer cells, suggesting a potential therapeutic value of targeting CDK1 for MYC-driven human breast cancer.

## Methods

### Cell lines, cell culture and reagents

The cell lines used in this study: AU565, BT20, BT474, BT483, BT549, HCC1143, HCC1500, HCC1569, HCC1937, HCC1954, HCC38, HCC70, Hs578T, MDA-MB-134, MDA-MB-175, MDA-MB-361, MDA-MB-436, MDA-MB-453, MDA-MB-468, SKBR3, and ZR751 were obtained from ATCC, Rockville, MD, USA. MCF-7 cells were obtained from Michigan Cancer Foundation, Detroit, MI, USA. The cell lines HBL100, MDA-MB-157, MDA-MB-231 and T47D were obtained from EG&G Mason Research Institute, Worcester, MA, USA.

The cell lines AU565, BT20, BT474, BT549, HBL100, Hs578T, MCF-7, MDA-MB-134, MDA-MB-157, MDA-MB-175, MDA-MB-231, MDA-MB-361, MDA-MB-436, MDA-MB-453, MDA-MB-468, SKBR3, T47D and ZR571 were cultured in RPMI 1640 medium supplemented with 10% heat-inactivated fetal bovine serum (FBS), 6 mM L-glutamine, 20 mM HEPES and 10 μg/ml human insulin (CSL-Novo, North Rocks, NSW, Australia). The remaining cell lines were cultured in RPMI 1640 medium supplemented with 10% heat-inactivated FBS, 6 mM L-glutamine, 1 mM sodium pyruvate and 20 mM HEPES. The MYC over-expressing MCF7 cells have been previously described [11,27] and were cultured in the same conditions as the parental cells.

The CDK4/6 inhibitor PD0332991 was purchased from Selleck Chemicals (Houston, TX, USA), CDK2 inhibitor SNS-032 from Symansis (Auckland, New Zealand) and CDK1 inhibitors, RO-3306 and CGP74514A, from Calbiochem (San Diego, CA, USA).

### Cell proliferation and apoptosis analysis

Bromodeoxyuridine (BrdU) incorporation was assayed using the Cell Proliferation ELISA, BrdU (colorimetric) Assay system (Roche, Dee Why, NSW, Australia). Proliferation was also assessed by AlamarBlue (Life technologies, Mulgrave, VIC, Australia). Cell cycle analysis was performed by flow cytometric analysis of propidium iodide-stained, ethanol-fixed cells. The apoptotic cell population was determined by staining methanol-fixed cells with the M30 CytoDEATH antibody (Enzo life Sciences, Farmingdale, NY, USA).

### siRNA transfection

The *MYC* siRNA pool contained equimolar concentrations of si*MYC*-17 (5′-GGACUAUCCUGCUGCCAAG-3′, Catalogue number D-003282-17-0050) purchased from Dharmacon (Lafayette, Colorado, USA) and *MYC* silencer (5′-GAGCUAAAACGGAGCUUUU-3′, Catalogue number s9130) purchased from Ambion (Austin, TX, USA). Gene-specific siRNAs including cyclin D1 (L-003210-00), *CDK2* (L-003236-00)), *CDK1* (J-003224-13) and On-Target Plus Non-Targeting siRNA control (D-001810-10), as well as siTOX transfection control (D-001500-01), were purchased from Dharmacon. siRNA transfection was performed by reverse transfection, where cells were seeded directly onto plates containing transfection reagents and siRNA mixture.

### Western blot analysis

Protein lysates were harvested as described previously [11]. 10 to 30 ug of lysate was separated using NuPage polyacrylamide gels (Life technologies, Mulgrave, VIC, Australia) prior to transfer to polyvinyl difluoride membranes. The membranes were incubated with the following primary antibodies: CDK1 (P34), CDK2 (M2), cyclin A (C-19), cyclin D1 (DCS-6), E2F1 (KH95) and MYC (9E10) from Santa Cruz Biotechnology (Santa Cruz, CA, USA); BIM, cyclin E1, cyclin E2, phospho-CDK2 (Thr160), phospho-pRB (ser795) (Cell Signaling, Danvers, MA, USA); p21^Cip1/Waf1^, p27^Kip1^ and pRB from BD Pharmingen (San Diego, CA, USA), β-Actin (AC15) from Sigma (St Louis, MO, USA). The secondary antibodies were horseradish peroxidase-conjugated sheep anti-mouse or donkey anti-rabbit antibodies (Amersham, Rydelmere, NSW, Australia), and specific proteins were visualized by chemiluminescence (Perkin-Elmer, Rowville, VIC, Australia). Densitometry was performed using the software ImageJ.

### Statistical analysis

All experiments were repeated at least three times. All numerical data are expressed as mean ± SEM. Statistical analyses were done by one-way ANOVA or linear regression using PRISM 6 (GraphPad, San Diego, CA). Error bars on all graphs represent the standard error of the mean between measurements. *P* < 0.05 was considered significant.

## Results

### SiRNA-mediated MYC knockdown identifies MYC-dependent breast cancer cells

**Figure 1:**
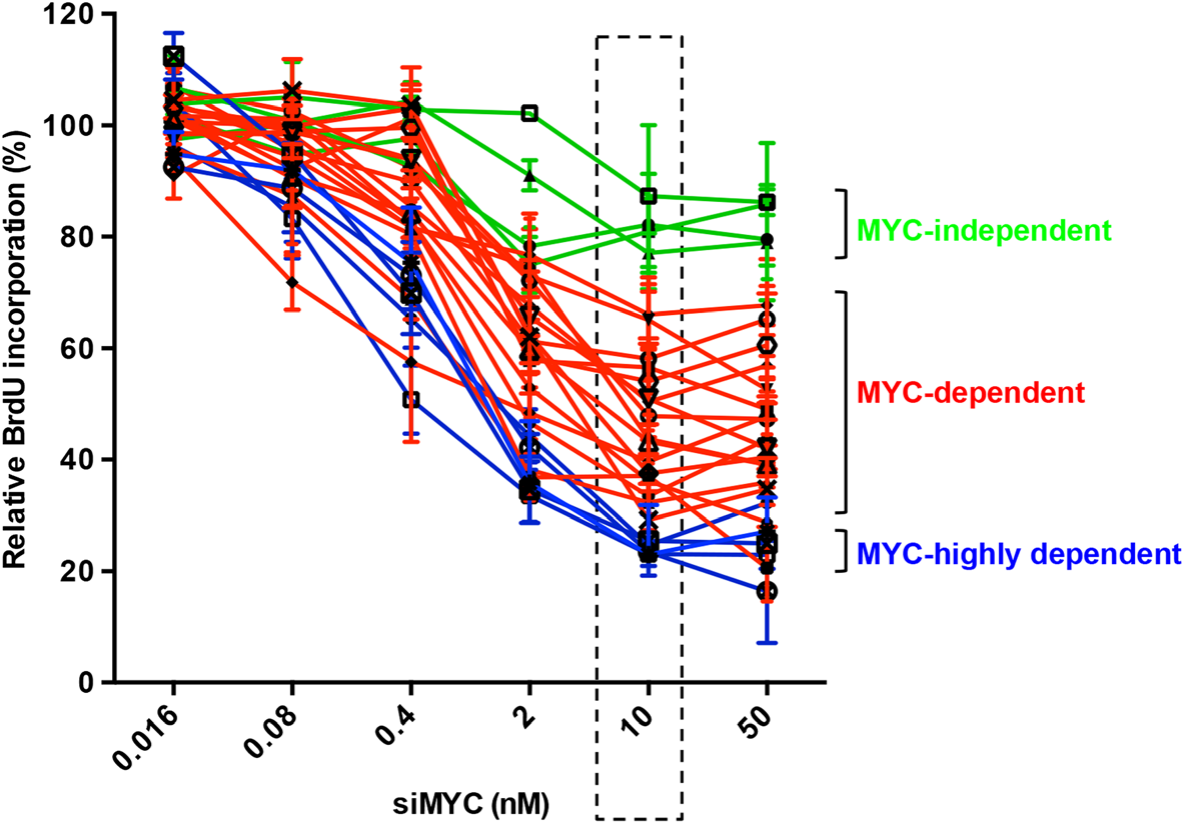
**The effects of siRNA-mediated MYC knockdown on cell proliferation in a panel of 26 human breast cancer cell lines.** Cells were transfected with either pooled MYC siRNA (siMYC) or Non-targeting siRNA control at the indicated concentrations and BrdU incorporation measured after 72 hours. Data are expressed as the percentage of BrdU incorporation in the cells transfected with MYC siRNA relative to the cells transfected with control siRNA. Based on their sensitivity to MYC depletion at 10 nM siRNA, breast cancer cells were classified into MYC-highly dependent (blue colour: Hs578T, HCC1954, MDA-MB-134, AU565 and SKBR3), dependent (red colour: HCC1143, BT549, BT474, MDA-MB-436. MDA-MB-231, MDA-MB-453, HCC70, T47D, MDA-MB-361, MCF-7, HBL100, ZR751, MDA-MB-468, BT483, HCC1937, HCC38 and HCC1569) and MYC-independent cells (green colour: MDA-MB-175, MDA-MB-157, BT20 and HCC1500) with inhibition of proliferation by more than 75%, 25-75% and less than 25%, respectively. Data are mean ± SEM analysed in triplicate experiments.

With the aim of assessing the dependence of human breast cancer cells on MYC function, we suppressed MYC expression through siRNA-mediated inhibition and then evaluated cellular proliferation. Twenty-six human breast cancer cell lines encompassing a spectrum of breast cancer phenotypes were transfected with a pool of two distinct siRNA species targeting different sequences within the *MYC* gene. For each cell line we tested different types of lipid transfection reagents and lipid concentrations, and titred the cell numbers plated for transfection (Additional file 1: Table S1). Optimized transfection conditions satisfied the following criteria: (1) Transfection with non-targeting siRNA control did not reduce cellular viability by more than 20%, compared to mock-transfected cells, indicating that these cells could be transfected without significant non-specific toxicity; (2) Transfection with a siRNA causing cellular toxicity led to a reduction in viability by more than 80%, compared to transfection with siCON, indicating that high transfection efficiency could be achieved. Western blot analyses confirmed that at a concentration of 10 nM siRNA, MYC expression was effectively decreased by 76.4% to 98.4% in all the cell lines tested (Additional file 1: Figure S1). BrdU incorporation was used a measure of the relative inhibition of cell proliferation in order to compare sensitivity to MYC knockdown across the panel of cell lines. As depicted in Figure 1, depletion of MYC decreased BrdU incorporation in a concentration-dependent manner in the majority of breast cancer cell lines, and the maximum inhibition occurred at 10–50 nM of MYC siRNA. However, the cell lines displayed a broad range of sensitivity to MYC RNAi in terms of the degree of inhibition achieved. The sensitivity of individual cell lines to MYC knockdown at 10 nM is listed in Additional file 1: Table S2. Transfection of 10 nM MYC siRNA resulted in a marked reduction of BrdU incorporation, by more than 75%, in five of twenty-six breast cancer cell lines (Hs578T, HCC1954, MDA-MB-134, AU565 and SKBR3). These cell lines were classified as highly MYC-dependent. BrdU incorporation was decreased by 50-75% in eleven cell lines (HCC1143, BT549, BT474, MDA-MB-436, MDA-MB-231, MDA-MB-453, HCC70, T47D, MDA-MB-361, MCF-7 and HBL100) and by 25-50% in six cell lines (ZR751, MDA-MB-468, BT483, HCC1937, HCC38 and HCC1569). In contrast, the BrdU incorporation of four cell lines (MDA-MB-175, MDA-MB-157, BT20 and HCC1500) was reduced by less than 25%, in the presence of 10 nM MYC siRNA. Since this group of cell lines was the least MYC-dependent, for simplicity we refer to them as MYC-independent.

### Sensitivity to siRNA-mediated MYC knockdown is correlated with MYC expression

**Figure 2:**
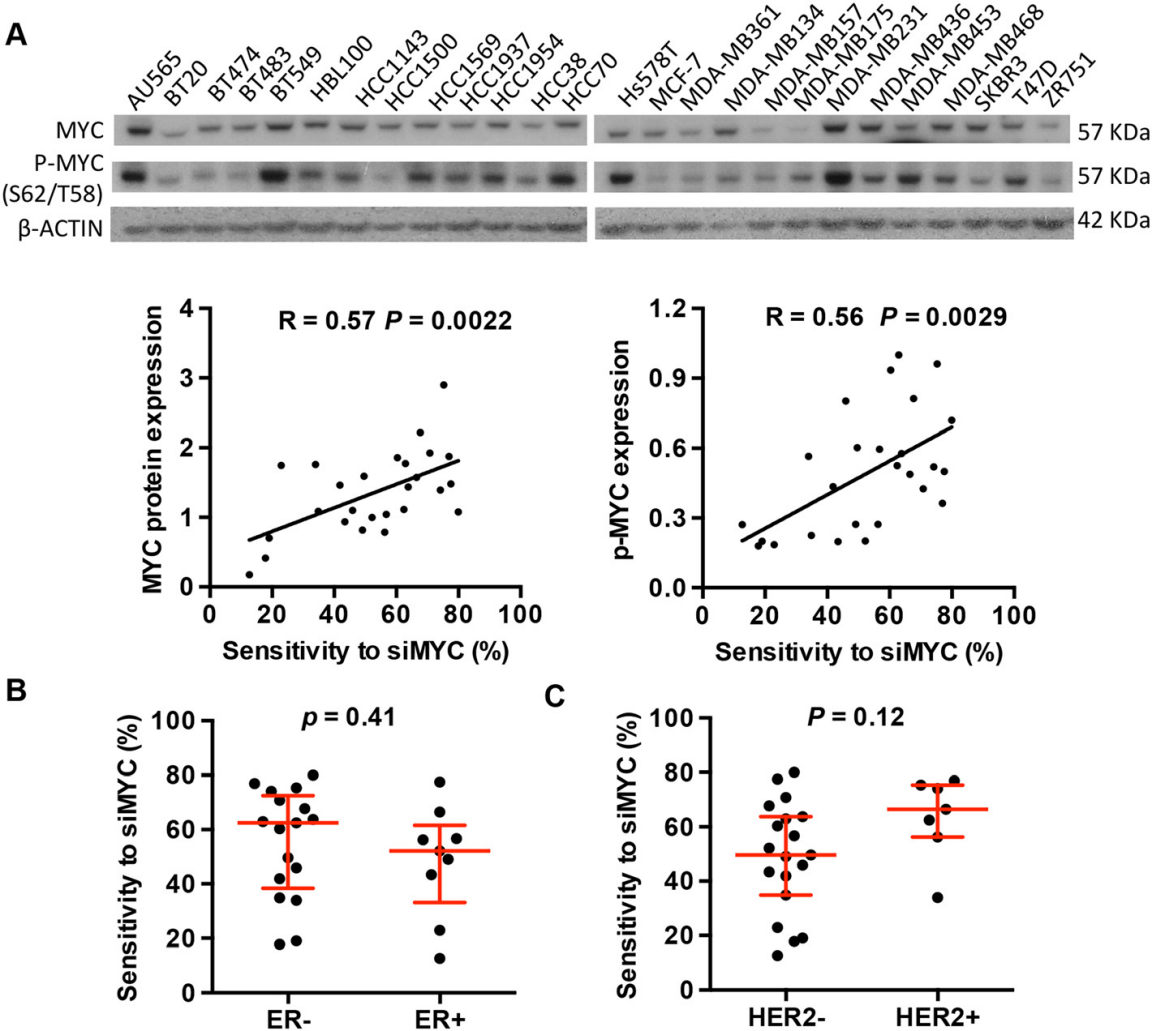
**Characterization of MYC dependence in human breast cancer cells. (A)** Protein expression levels of MYC and phospho-MYC at T58 and S62 in a panel of 26 human breast cancer cell lines were analysed by Western blotting and quantitated by densitometry. The correlation between sensitivity to MYC siRNA (siMYC) and MYC protein expression level or MYC phosphorylation level was assessed. Sensitivity to MYC siRNA was determined by inhibition of BrdU incorporation after MYC depletion at 10 nM siRNA for each cell line in the panel and summarized in Additional file 1: Table S2. Higher numbers indicate higher sensitivity, i.e. sensitivity to MYC siRNA = 100% - relative BrdU incorporation. Sensitivity to MYC RNAi across ER- and ER + subtypes **(B)**, and HER2- and HER2+ subtypes **(C)** were compared. R = Pearson correlation coefficient, *P* = the corresponding *P*-value.

To characterize the molecular features of MYC-dependent breast cancer cells, we analysed the baseline MYC signalling activity. Sensitivity to MYC knockdown (i.e. 100% minus the relative BrdU incorporation) was significantly correlated with MYC mRNA (R = 0.59, *P* = 0.0014, Additional file 1: Figure S2A) and protein expression level (R = 0.57, *P* = 0.0022, Figure 2A and Additional file 1: Table S2). Phosphorylation of MYC at threonine 58 and serine 62 is critical for stabilization of MYC protein [28], and there was a significant correlation between MYC phosphorylation status and responsiveness to MYC depletion (R = 0.56, *P* = 0.0029, Figure 2A and Additional file 1: Table S2). Although no overall correlation was observed between MYC gene copy number and sensitivity to MYC siRNA, two cell lines with *MYC* gene amplification, AU565 and SKBR3, displayed a high sensitivity to MYC depletion (Additional file 1: Figure S2B). In addition, there was no significant correlation between baseline expression of the cell cycle effector proteins, cyclin D1, cyclin E1 and E2, cyclin A, CDK2 and the CDK inhibitors (p21, p27 and p16), and sensitivity to MYC siRNA (Additional file 1: Figure S2C). We also noted that the inhibitory effect of MYC siRNA on cell proliferation was not dependent on retinoblastoma protein (pRb) status, as five pRb-deficient cell lines (BT549, MDA-MB-436, HCC70, MDA-MB-468 and HCC1937) were all responsive to MYC depletion, implicating both pRb-dependent and pRb-independent mechanisms underlying MYC-stimulated cell cycle progression.

Breast cancer cell lines have been classified into three broad subtypes based on the expression of the intrinsic genes: luminal, basal A or basal B [29,30]. There was no significant correlation between molecular subtype and sensitivity to MYC knockdown (Additional file 1: Table S4). Although the median value of sensitivity of ER-negative breast cancer cells was higher than that of ER-positive cells, the difference between these two subtypes did not reach statistical significance (Figure 2B). Similarly, although all seven *HER2*-amplified cell lines were MYC-dependent, and as a group had a tendency to exhibit higher sensitivity to MYC knockdown than non-*HER2* amplified cell lines (Figure 2C), there was no statistically significant difference between *HER2*-amplified and non-*HER2* amplified cells.

### MYC-dependent breast cancer cells are not sensitive to CDK4/6 inhibition

**Figure 3:**
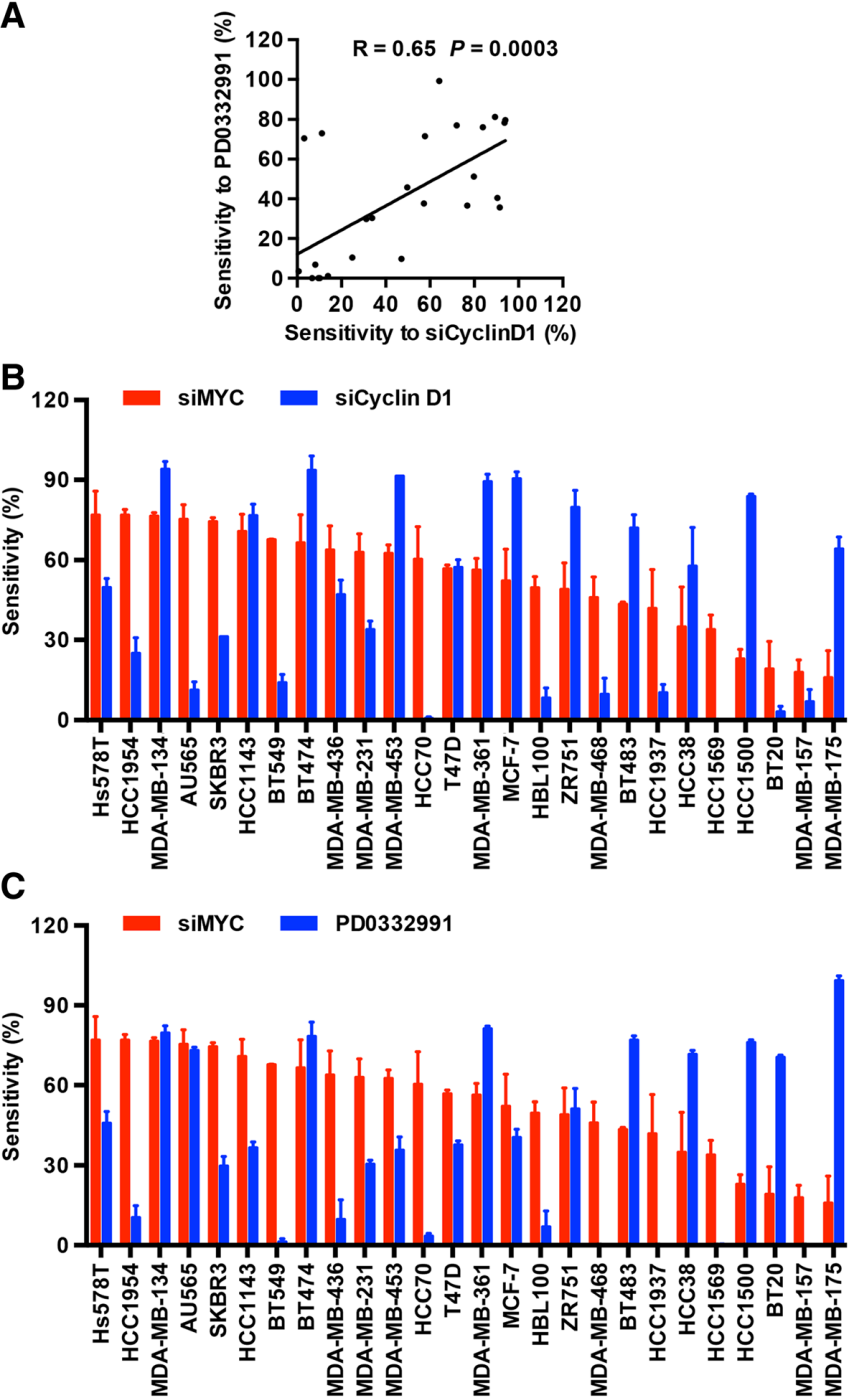
**MYC-dependent breast cancer cells are not selectively sensitive to CDK4/6 inhibition.** A panel of 26 human breast cancer cell lines were transfected with either pooled cyclin D1 siRNA (siCyclin D1) or Non-targeting siRNA control for 3 days or treated with CDK4/6 inhibitor PD0332991 for 2 days and cell proliferation measured by BrdU incorporation. Sensitivity to cyclin D1 siRNA, MYC siRNA or PD0332991 was determined by inhibition of BrdU incoporation at 2 nM cyclin D1 siRNA, 10 nM MYC siRNA or 0.25 μM PD0332991, respectively, for each cell line in the panel and summarized in Additional file 1: Table S2 and S3. Higher numbers indicate higher sensitivity. **(A)** The correlation between sensitivity to cyclin D1 knockdown and PD0332991. **(B)** The correlation between sensitivity to MYC siRNA and cyclin D1 siRNA. **(C)** The correlation between sensitivity to MYC siRNA and PD0332991. Data are mean ± SEM analysed in triplicate in duplicate experiments. R = Pearson correlation coefficient, *P* = the corresponding *P*-value.

CDK4/6 inhibition has been proposed as a therapeutic strategy in breast cancer, particularly in ER-positive breast cancer [31,32]. To determine the relationship between dependence on CDK4/6 activity and MYC dependence in breast cancer cells, a panel of twenty-six breast cancer cell lines was treated with either cyclin D1 siRNA or the CDK4/6 inhibitor PD0332991 (Additional file 1: Figure S3). Western blot analyses confirmed that at a concentration of 2 nM siRNA cyclin D1 expression was substantially decreased in all the cell lines tested (Additional file 1: Figure S3A). Consistent with the finding by Finn and colleagues [32], ER-positive cells were more sensitive to cyclin D1 knockdown and PD0332991 treatment than ER-negative cells. Eight cell lines demonstrated resistance to both cyclin D1 knockdown and PD0332991 (< 25% inhibition). Although two cell lines (BT20 and AU565) were relatively insensitive to cyclin D1 siRNA (< 25% inhibition) but sensitive to PD0332991, and one cell line, MDA-MB-436, was resistant to PD0332991 but showed response to cyclin D1 knockdown (Additional file 1: Table S3), overall sensitivity to PD0332991 was significantly correlated with sensitivity to cyclin D1 knockdown (R = 0.65, *P* = 0.0003, Figure 3A), confirming cyclin D1 as a major activator of CDK4/6 in regulation of breast cancer cell cycle progression.

Subsequently, cell sensitivity to MYC depletion and sensitivity to cyclin D1 or CDK4/6 inhibition were compared (Figure 3). Overall no significant correlation was observed between the responsiveness to MYC RNAi and cyclin D1 RNAi (Figure 3B) or MYC RNAi and PD0332991 (Figure 3C). Interestingly, eight of nine PD0332991-resistant cell lines were responsive to MYC depletion while three of four MYC-independent cell lines were sensitive to PD0332991.

### CDK2 inactivation accompanies cell cycle arrest by MYC depletion but is not selectively lethal to MYC-dependent breast cancer cells

**Figure 4:**
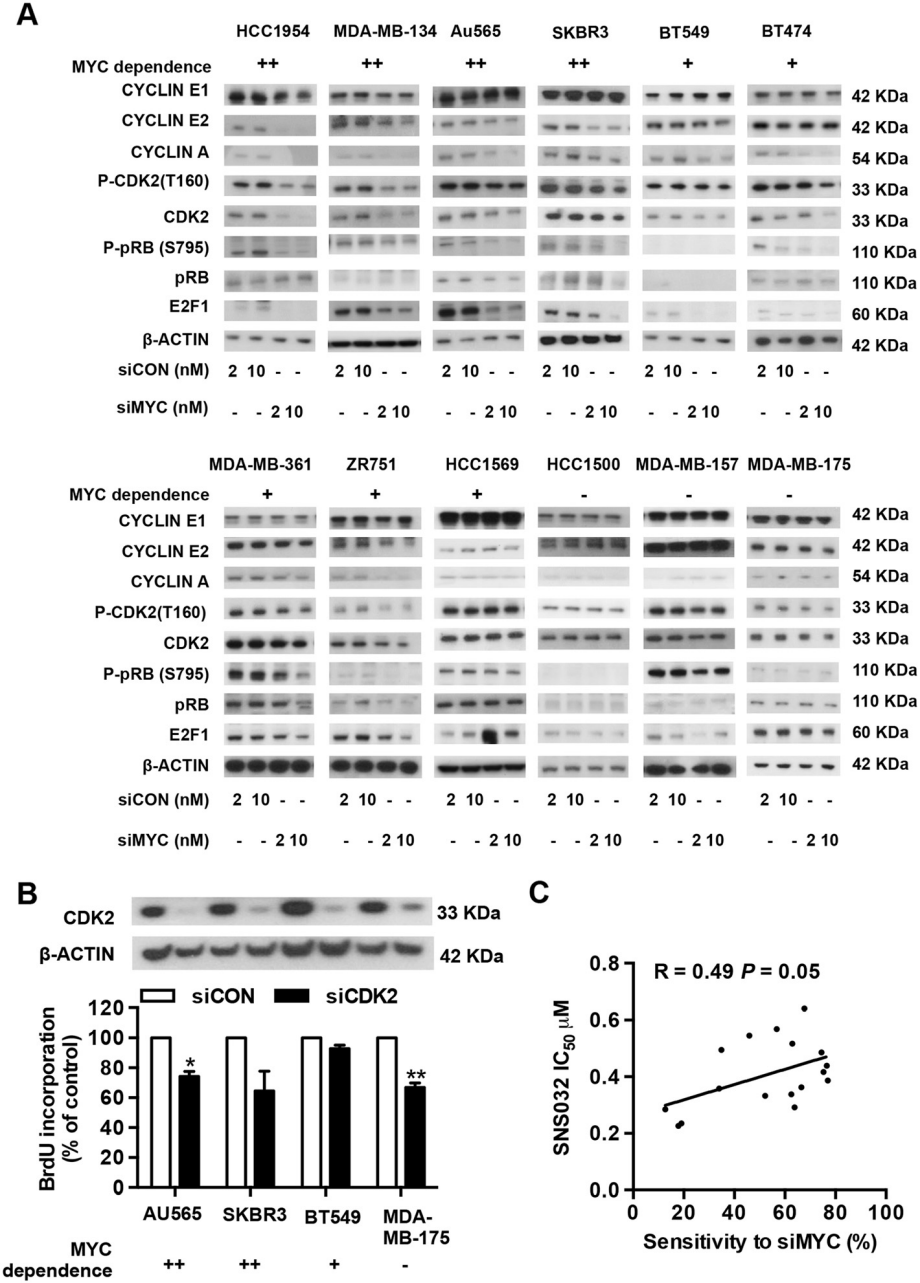
**CDK2 inactivation mediates cell cycle arrest by MYC depletion and is not selectively lethal to MYC-dependent breast cancer cells. (A)** Cells were transfected with either pooled MYC siRNA (siMYC) or Non-targeting siRNA control (siCON) at 2 nM or 10 nM. Cell lysates were collected at 72 hour post-transfection and then subjected to Western blotting. β-ACTIN was used as loading control for whole-cell lysates. **(B)** Cells were transfected with either pooled CDK2 siRNA (siCDK2) or Non-targeting siRNA control (siCON) at 10 nM. 72 hours after transfection, cell proliferation was measured by BrdU incorporation. Data are mean ± SEM analysed in triplicate experiments. **(C)** A panel of 17 human breast cancer cell lines were treated with CDK2 inhibitor SNS-032 at a series of concentrations for 48 hours followed by measurement of BrdU incorporation. The correlation between sensitivity to MYC siRNA and SNS-032 was assessed. Sensitivity to MYC siRNA was determined by inhibition of BrdU incorporation at 10 nM MYC siRNA and sensitivity to SNS-032 determined by the IC_50_ value for each cell line shown in Additional file 1: Table S2 and S3, respectively. R = Pearson correlation coefficient, *P* = the corresponding *P*-value.

Since CDK2 activity is a principal target for MYC stimulation of G_1_-S progression, we investigated whether differences in CDK2 activity might account for the differential effects of MYC depletion in breast cancer cells. We assessed CDK2 activity using phosphorylation of CDK2 at threonine 160 [33] in MYC-dependent and MYC-independent cells. Western blot analyses demonstrated that CDK2 activity was decreased in 7 of 9 MYC-dependent cells (HCC1954, MDA-MB-134, AU565, SKBR3, BT474, MDA-MB-361 and ZR751), but not in MYC-independent cells (HCC1500, MDA-MB-157 and MDA-MB-175), after MYC knockdown (Figure 4A). CDK2 is activated by binding of cyclin E or cyclin A. Although MYC depletion did not affect cyclin E1 expression, cyclin E2 expression was reduced by MYC RNAi in 5 of 9 MYC-dependent cell lines (HCC1954, MDA-MB-134, Au565, SKBR3 and ZR751). Similarly, except HCC1569, 8 of 9 MYC-dependent cell lines displayed down-regulation of cyclin A expression upon MYC depletion, whereas there was no change of cyclin A expression in MYC-independent cell lines. During the G_1_-S phase transition, pRb is successively phosphorylated by cyclin D1-CDK4/6 and cyclin E/A-CDK2 complexes, resulting in E2F1 activation and the expression of E2F target genes that promote cell cycle progression [34]. Analysis of the phosphorylation status of pRb at serine 795, a site targeted by CDK2 and CDK4 [35], revealed that there was a sustained decrease in pRb phosphorylation in MYC-dependent cells after MYC knockdown. Accordingly, a significant decrease of E2F1 expression was observed after MYC siRNA treatment. Interestingly, in BT549 cells, which have undetectable pRb expression and pRb phosphorylation, E2F1 expression was still reduced following MYC knockdown (Figure 4A). In contrast, three MYC-independent cell lines did not show a significant decrease in either pRb phosphorylation or E2F1 expression following MYC knockdown (Figure 4A). These observations indicate that in MYC-dependent cells, a decrease in CDK2 activity is a common event upon MYC depletion, and leads to a subsequent decrease of pRb phosphorylation and E2F1 activity, contributing to MYC RNAi-induced cell cycle arrest.

Given the relationship between MYC dependence and CDK2 activity, we therefore assessed whether targeting CDK2 could block the proliferation of three MYC-dependent breast cancer cell lines. In SKBR3 and AU565, siRNA-mediated CDK2 knockdown decreased BrdU incorporation by 35.4% and 25.8%, respectively, whereas the relative BrdU incorporation in BT549 cells only dropped by 7.2%. Strikingly, the MYC-independent cell line, MDA-MB-175, showed a 33.2% decrease in BrdU incorporation upon CDK2 depletion (Figure 4B). CDK2 siRNA did not induce cell apoptosis in any of the cell lines tested (data not shown), which was in agreement with the previous observation of lack of cell death after CDK2 inhibition in several cancer cell types [36]. In contrast to the modest inhibitory effect of CDK2 knockdown on cell cycle progression, SNS-032, a CDK inhibitor with relatively high affinity for CDK2, 7 and 9 [37], caused a marked inhibition of cell proliferation (Additional file 1: Figure S4). Surprisingly, an inverse correlation between sensitivity to MYC RNAi and sensitivity to SNS-032 was observed (R = 0.49, *P* = 0.05, Figure 4C), i.e. MYC-dependent cells are likely to be more resistant to SNS-032 than MYC-independent cells.

### CDK1 inhibition selectively reduces viability of MYC-dependent cells

**Figure 5:**
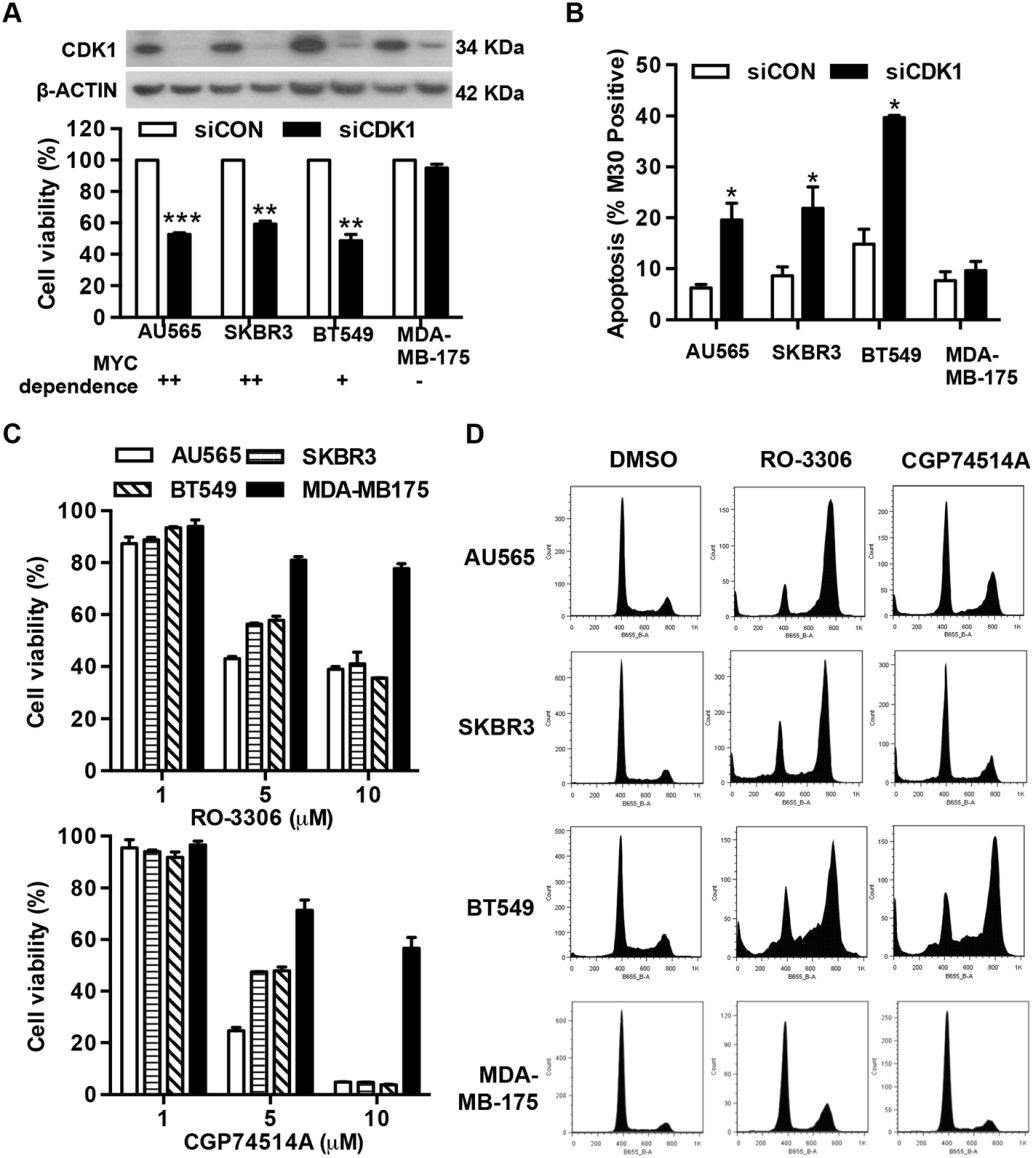
**CDK1 inhibition selectively reduced viability of MYC-dependent breast cancer cells. (A)** Cells were transfected with either pooled CDK1 siRNA (siCDK1) or Non-targeting siRNA control (siCON) at 10 nM. Cell viability was measured by AlamarBlue assay 5 days post-transfection. **(B)** Cell apoptosis was assessed by the detection of caspase-cleaved cytokeratin 18 with M30 cytoDEATH™ using flow cytometry. **(C)** Cells were treated with CDK1 inhibitor RO-3306 or CGP74514A at the indicated concentrations for 3 days. Cell viability was measured by AlamarBlue assay and the data expressed as the percentage of viability relative to vehicle-treated cells. **(D)** Representative histograms 48 hours after treatment with RO-3306 or CGP74514A at 5 μM. *P < 0.05, **P < 0.01, ***P < 0.001, compared with cells transfected with control siRNA.

We next investigated whether MYC-dependent cells are more sensitive to CDK1 inhibition than MYC-independent cells. As shown in Figure 5A, siRNA-mediated CDK1 depletion significantly reduced cell viability in the three MYC-dependent breast cancer cell lines AU565, SKBR3 and BT549, but did not affect cell viability of the MYC-independent cell line MDA-MB-175. Moreover, CDK1 depletion increased cell apoptosis by 2.2-fold in AU565, 2.3-fold in SKBR3 and 3.1-fold in BT549 cells, but did not induce apoptosis in MDA-MB-175 cells (Figure 5B). Cell cycle profiles revealed that siRNA-mediated CDK1 knockdown blocked the cell cycle in the G_2_/M phase and induced apoptosis in AU565, SKBR3 and BT549 cells but did not significantly affect cell cycle progression in MDA-MB-175 cells (Additional file 1: Figure S5A). Similar results were obtained by using two structurally distinct CDK1 inhibitors, RO-3306 [38] and CGP74514A [39]. MYC-independent cells MDA-MB-175 were more resistant to cell death (Figure 5C) and cell cycle arrest (Figure 5D) induced by RO-3306 and CGP74514A than three MYC-dependent cell lines, indicative of reduced sensitivity to CDK1 inhibition in MYC-independent cells.

### CDK1 inhibitor-induced cell apoptosis is MYC-dependent

**Figure 6:**
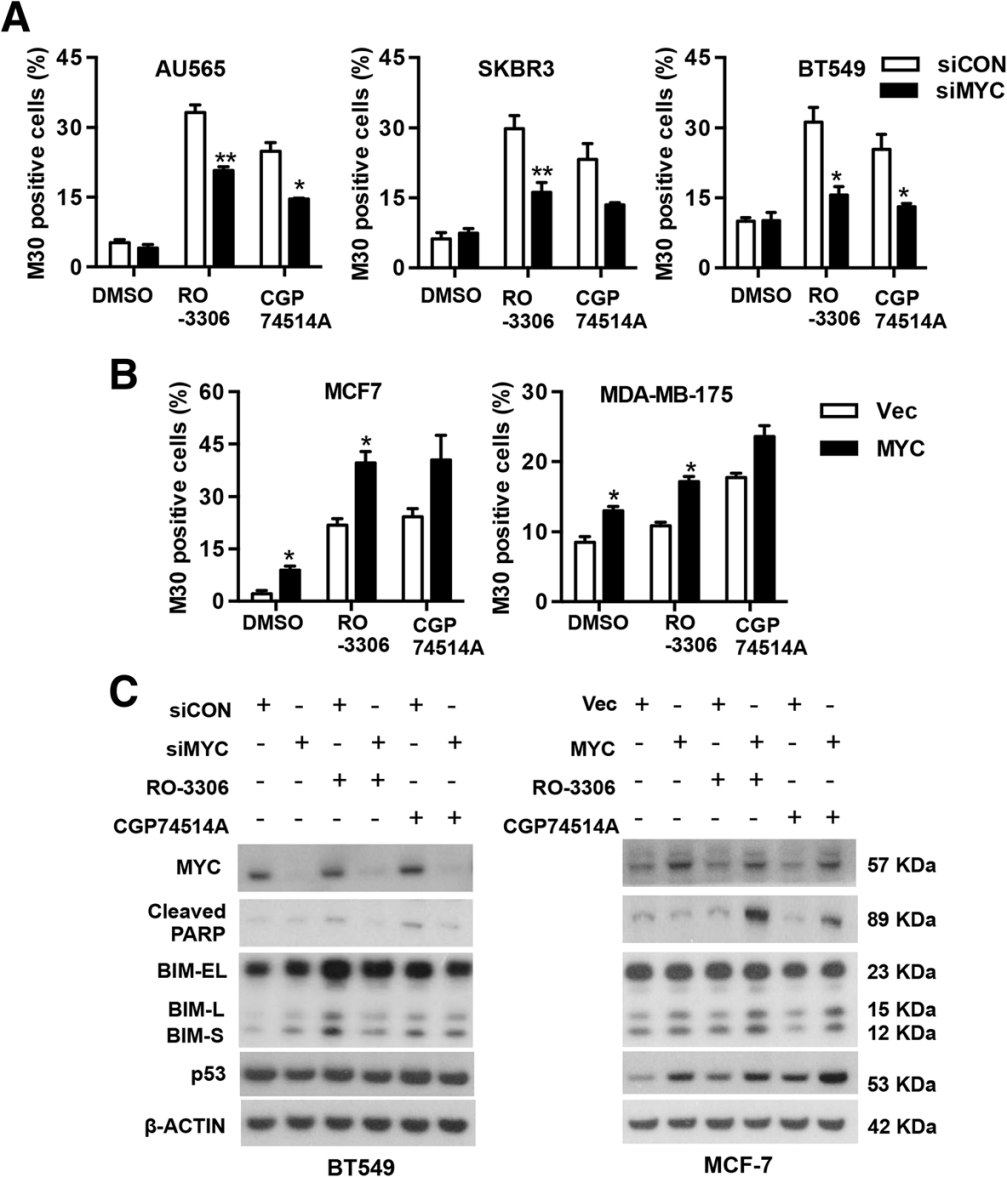
**CDK1 inhibitor-induced apoptosis in human breast cancer cells is MYC-dependent. (A)** Cells transfected with either pooled MYC siRNA (siMYC) or Non-targeting siRNA control (siCON) at 10 nM were treated with CDK1 inhibitor RO-3306 or CGP74514A at 5 μM for 48 hours. Cell apoptosis was assessed by the detection of caspase-cleaved cytokeratin 18 with M30 cytoDEATH™ using flow cytometry. **(B)** MCF-7 cells and MDA-MB-175 cells stably transfected with MYC expressing plasmids (MYC) or the vector control (Vec) were treated with either RO-3306 or CGP74514A at 5 μM for 48 hours and cell apoptosis measured by M30 cytoDEATH™ in flow cytometry. **(C)** BT549 cells transfected with siMYC or siCON (left panel), and MCF-7 cells over-expressing MYC and the control cells (right panel) were treated with either RO-3306 or CGP74514A at 5 μM for 48 hours. The whole cell lysates were harvested and subjected to Western blotting. *P < 0.05, **P < 0.01, compared with cells transfected with control siRNA for the same treatment.

To investigate whether the observed selective CDK1-induced cell death was dependent on MYC status, we suppressed MYC expression by transfection of MYC siRNA in three MYC-dependent cells (AU565, SKBR3 and BT549 cells) and subsequently treated cells with the CDK1 inhibitor RO-3306 or CGP74514A. Depletion of MYC decreased RO-3306-induced cell apoptosis by 12.5%, 13.7% and 15.7%, and CGP74514A-induced cell apoptosis by 10.3%, 10.8% and 12.3% in AU565, SKBR3 and BT549 cells, respectively (Figure 6A). Furthermore, we stably transfected MCF-7 cells and MDA-MB-175 cells with a plasmid expressing MYC or a control plasmid and then measured the effect of MYC over-expression on cell response to RO-3306 and CGP74514A. As expected, increased cell apoptosis was observed in the presence of CDK1 inhibitor upon MYC over-expression (Figure 6B), suggesting CDK1 inhibitor-induced cell apoptosis is partially MYC-dependent.

To determine how the CDK1 inhibitor induced cell apoptosis in MYC-dependent cells, we examined the protein expression of components of the intrinsic apoptosis pathway in BT549 cells transfected with either MYC siRNA or control siRNA, followed by treatment with RO-3306 or CGP74514A. The protein expression level of BIM, a pro-apoptotic BCL-2 family member, was up-regulated in cells treated with CDK1 inhibitors, and cells transfected with MYC siRNA showed lower BIM expression than cells transfected with control siRNA in the presence of CDK1 inhibitors (Figure 6C left panel). Similar results were also observed in the other two MYC-dependent cell lines AU565 and SKBR3 (Additional file 1: Figure S5B). Conversely, increased BIM expression was observed in MCF-7 cells (Figure 6C right panel) and MDA-MB-175 cells (Additional file 1: Figure S5B) over-expressing MYC in response to CDK1 inhibitors compared to the control cells, supporting a functional role of BIM in CDK1 inhibitor-induced apoptosis in MYC dependent-cells. The expression level of another pro-apoptotic protein, p53, was also up-regulated in AU565 and SKBR3 cells treated with CDK1 inhibitors and MYC knockdown prevented increase of p53 expression by CDK1 inhibitors (Additional file 1: Figure S5B), whereas MYC overexpression in MCF-7 cells (Figure 6C right panel) and MDA-MB-175 cells (Additional file 1: Figure S5B) induced p53 expression upon treatment with CDK1 inhibitors. Notably, in BT549 cells which carry a mutant *p53* gene, p53 expression in either MYC-depleted cells or the control cells was not affected by CDK1 inhibitors (Figure 6C left panel), suggesting that CDK1 inhibitor-induced apoptosis in MYC-dependent cells is p53-independent.

## Discussion

MYC is a pivotal regulator of cell growth in breast cancer [27]. In transgenic mouse models with inducible MYC, withdrawal of MYC expression induces breast tumour regression, indicating these tumours are addicted to MYC function for tumour maintenance [40-42]. We herein exploited this oncogenic addiction to assess the dependence of human breast cancer cells on MYC function through an RNAi approach. Depletion of MYC blocked proliferation of 85% of breast cancer cell lines (22/26 cell lines), which were classified as MYC-dependent breast cancer cells while 15% of cell lines (4/26 cell lines) showed resistance to MYC RNAi and were therefore classified as MYC-independent cells. We further identified that MYC-dependent breast cancer cells possessed high MYC protein expression and high MYC phosphorylation level, suggesting an elevated MYC signalling activity in these cells. Through identification of a MYC transcription gene signature, several studies have uncovered an enrichment of MYC-driven transcription programs in basal breast cancer [43-45]. More recently, Horiuchi *et al.* reported that triple negative breast cancer exhibited increased activity of the MYC pathway [46]. However, we did not found a significant difference between ER + and ER- cells in MYC dependence, although ER- cells tend to be more sensitive to MYC depletion than ER + cells. Instead, our data showed that all *HER2*-amplified cell lines were dependent on MYC function, raising the possibility of a biological link between MYC and HER2 pathways. As a downstream target of HER2 signalling, MYC mediates HER2-driven proliferative activity in breast cancer cells [47]. *MYC* amplification has also been significantly associated with *HER2* amplification in human breast tumours [48]. Although patients with *MYC/HER2* co-amplified breast tumours have worse outcomes than patients with single gene amplified tumours [49], the predictive value of *MYC* amplification in the response to adjuvant trastuzumab in HER2-positive breast tumours is still unclear [50]. Nevertheless, our finding implicated a therapeutic potential of MYC inhibition in *HER2*-amplified breast cancers.

Since directly targeting MYC remains a challenge in clinical practice, either targeting components of the MYC pathway, or using a synthetic lethal strategy have been suggested as new options for MYC-dependent malignancies [17]. In this study we assessed the therapeutic potential of specific CDK inhibition in MYC-dependent breast cancer cells. *CDK4* has been identified as a key MYC target gene in mammals [16]. *CDK4*-deficient mice were resistant to skin tumour development induced by MYC [15], whereas mice lacking cyclin D1 expression and consequently lacking CDK4 activation still developed mammary tumours induced by MYC activation [51], strongly arguing that the requirement for CDK4 activity in MYC-induced tumorigenesis is affected by cellular context and tissue type. In agreement with *in vivo* observations, our study identified a distinct response pattern to MYC inhibition compared to cyclin D1-CDK4/6 inhibition in breast cancer cells. Studies here and by others [32] demonstrated that cells with luminal ER-positive subtype and a functional pRb pathway were more sensitive to cytostatic effects of cyclin D1 and CDK4/6 inhibition. In contrast, the response to MYC depletion was not dependent on pRb status and was not significantly correlated with the molecular subtypes present in the panel of cell lines. Overall, our data suggested that CDK4/6 inhibitors are unlikely to be useful in the treatment of MYC-dependent breast tumours.

Activation of CDK2, another interphase CDK, is involved in MYC regulation of G_1_-S phase transition. As a major event downstream of MYC activation, CDK2 activation can also suppress MYC-induced senescence [52], which raised the possibility of CDK2 as a potential therapeutic target for MYC-dependent cancers. Consistent with our previous results [11,27], this study demonstrated that depletion of MYC reduced CDK2 activity in MYC-dependent cells but not in MYC-independent cells, indicative of a role of CDK2 inactivation in MYC inhibition-induced cell cycle arrest. This occurred in the absence of changes of cyclin E1 expression. Despite our previous study showing cyclin E2 was up-regulated through cyclin D1 but not MYC in MCF-7 cells [53], we noted that cyclin E2 expression was reduced following MYC RNAi in 5 of 9 MYC-dependent cell lines, suggesting cell type and genetic context-dependent regulation of cyclin E2 expression in breast cancer cells. Although CDK2 inactivation has been reported to induce apoptosis in MYCN-amplified neuroblastoma cells [21], in this study, siRNA-mediated CDK2 depletion failed to induce apoptosis in breast cancer cells. Inhibition of CDK2 by either siRNA or an inhibitor, reduced cell proliferation of both MYC-independent and MYC-dependent cells, and MYC-independent cells possessed relatively high sensitivity to a CDK2 inhibitor, SNS-032. Therefore, our data do not support a synthetic lethal interaction between CDK2 inactivation and MYC activation in breast cancer cells.

Unlike CDK4, CDK6 and CDK2 which are redundant for the mammalian cell cycle, CDK1 is essential for cell division and sufficient for driving the cell cycle in all cell types [25,54]. CDK1 regulates chromosome condensation and microtubule dynamics to facilitate the transition from G_2_ to M phase. Goga *et al.* reported that CDK1 inhibition resulted in a synthetic lethality in mouse lymphoma and hepatoblastoma with MYC hyper-activation [22]. We showed here that breast cancer cells were also selectively sensitive to CDK1 inhibition. The different sensitivity did not appear to be related to CDK1 expression, since CDK1 expression did not vary markedly between the cell lines used here, consistent with previous data showing that there are not large variations in CDK1 expression in breast cancer cell lines [55]. Instead, the sensitivity to CDK1 inhibition appeared to reflect a synthetic lethal interaction between MYC and CDK1 [22,46]. One potential mechanism for this synthetic lethal interaction is that loss of CDK1 leads to substantial mitotic catastrophe [56], which possibly increases MYC-induced replication stress, and subsequently activates checkpoint signalling, resulting in cell death. Thus cells harbouring MYC hyper-activation might be more vulnerable to mitotic disruption. Indeed, several MYC synthetic lethal genes identified in recent studies, including aurora-B kinase, CHK1/2 and SUMO-activating enzyme, are all involved in maintaining mitotic fidelity [20,23,57]. Moreover, high-throughput screens display enrichment of the components of the mitotic spindle among MYC synthetic lethal candidates [19,20]. Therefore, specific targeting of CDK1 might be effective for breast tumours dependent on MYC activation and this synthetic lethal strategy may overcome some problems with side effects induced by CDK1 inhibition.

Previous studies showed that up-regulation of BIM expression was required for MYC overexpression-induced apoptosis [58] and contributed to cell death induced by the CDK inhibitor purvalanol A in breast cancer cells [46]. Consistent with these studies, we found that CDK1 inhibitors induced BIM expression and that elevated BIM expression was associated with increased sensitivity to CDK1 inhibitors in cells with high MYC expression. p53, however, appears to be dispensable for increased cell apoptosis induced by CDK1 inhibitors, although loss of p53 has been reported to reduce cell apoptosis associated with MYC overexpression [59]. p53-independent apoptosis was also observed in MYC-overexpressing mouse embryo fibroblast cells treated with purvalanol A [22]. Therefore, specific apoptotic pathways appear to be involved in CDK1 inhibitor-induced MYC-dependent cell death, providing a mechanistic insight into MYC-CDK1 synthetic lethality in breast cancer cells.

## Conclusions

This study identified that MYC-dependent breast cancer cells possess high MYC expression and high level of MYC phosphorylation, and are sensitive to inhibition of CDK1, but not CDK4/6 and CDK2, suggesting that high MYC expression in breast cancer cells is associated with selective synthetic lethality induced by CDK1 inhibition. Since understanding the role of individual CDK activities in specific tumour subtypes is essential to improve efficacy of CDK inhibitors in clinical practice, selective inhibition of CDK1 warrants further investigation as a potential therapy for MYC-dependent breast cancers.

## Abbreviations

CDK: Cyclin-dependent kinase; siRNA: Small interfering RNA.

## Competing interests

The authors declared that they have no competing interests.

## Authors’ contributions

JK designed the study, carried out the experiments, analysed the data, drafted the manuscript and read and approved the final manuscript. CMS carried out the experiments and read and approved the final manuscript. RLS conceived of the study, and participated in its design and coordination. EAM conceived of the study, participated in its design and coordination, helped to draft the manuscript and read and approved the final manuscript.

## Pre-publication history

The pre-publication history for this paper can be accessed here:

http://www.biomedcentral.com/1471-2407/14/32/prepub

## Supplementary Material

Additional file 1 **Kang et al., Targeting cyclin-dependent kinase 1(CDK1) but not CDK4/6 or CDK2 is selectively lethal to MYC-dependent human breast cancer cells. Figure S1.** Western blot analyses to show efficiency of siRNA-mediated inhibition of MYC expression in a panel of 26 human breast cancer cell lines. **Figure S2.** The correlation of sensitivity to MYC siRNA to *MYC* mRNA expression or *MYC* copy number. **Figure S3.** The effects of cyclin D1 or CDK4/6 inhibition on cell proliferation in a panel of 26 human breast cancer cell lines. **Figure S4.** The effects of CDK2 inhibitor SNS-032 on cell proliferation in a panel of 17 human breast cancer cell lines. **Figure S5.** The effects of CDK1 inhibition on cell cycle progression and cell apoptosis in human breast cancer cells. **Table S1.** Optimised transfection conditions in a 96-well plate format for human breast cancer cell lines. **Table S2.** The sensitivity of human breast cancer cell lines to siRNA-mediated inhibition of MYC and MYC protein expression in breast cancer cells. **Table S3.** The sensitivity of human breast cancer cell lines to siRNA-mediated inhibition of cyclin D1expression, PD0332991 and SNS-032. **Table S4.** Molecular features of human breast cancer cell lines. Click here for file
