# Supplementary material for: Targeting cyclin-dependent kinase 1 (CDK1) but not CDK4/6 or CDK2 is selectively lethal to MYC-dependent human breast cancer cells
Source: BMC Cancer. 2014 Jan 20;14:32. doi: 10.1186/1471-2407-14-32 (PMC3903446; doi:10.1186/1471-2407-14-32)
Supplement: Additional file 1 — Kang et al., Targeting cyclin-dependent kinase 1(CDK1) but not CDK4/6 or CDK2 is selectively lethal to MYC-dependent human breast cancer cells. Figure S1. Western blot analyses to show efficiency of siRNA-mediated inhibition of MYC expression in a panel of 26 human breast cancer cell lines. Figure S2. The correlation of sensitivity to MYC siRNA to MYC mRNA expression or MYC copy number. Figure S3. The effects of cyclin D1 or CDK4/6 inhibition on cell proliferation in a panel of 26 human breast cancer cell lines. Figure S4. The effects of CDK2 inhibitor SNS-032 on cell proliferation in a panel of 17 human breast cancer cell lines. Figure S5. The effects of CDK1 inhibition on cell cycle progression and cell apoptosis in human breast cancer cells. Table S1. Optimised transfection conditions in a 96-well plate format for human breast cancer cell lines. Table S2. The sensitivity of human breast cancer cell lines to siRNA-mediated inhibition of MYC and MYC protein expression in breast cancer cells. Table S3. The sensitivity of human breast cancer cell lines to siRNA-mediated inhibition of cyclin D1expression, PD0332991 and SNS-032. Table S4. Molecular features of human breast cancer cell lines. [file 1471-2407-14-32-S1.pdf]

Additional File

Kang et al, Targeting cyclin-dependent kinase 1(CDK1) but not CDK4/6 or CDK2 is selectively lethal to MYC-dependent human breast cancer cells.

Figure S1 Western blot analyses to show efficiency of siRNA-mediated inhibition of MYC expression in a panel of 26 human breast cancer cell lines. Cells were transfected with either pooled MYC siRNA (siMYC) or Non-targeting siRNA control (siCON) at 2 nM or 10 nM. Cell lysates were collected after 72 hours and subjected to Western blotting for MYC. β-ACTIN was used as loading control for whole-cell lysates.

Figure S2 The correlation of sensitivity to MYC siRNA to *MYC* mRNA expression or *MYC* copy number. Sensitivity to MYC siRNA was determined by inhibition of BrdU incorporation by 10 nM MYC siRNA shown in Additional file Table S2. Higher numbers indicate higher sensitivity. (C) The expression of cell cycle-related proteins in a panel of 26 breast cancer cell lines. β-ACTIN was used as loading control for whole-cell lysates.

Figure S3 The effects of cyclin D1 or CDK4/6 inhibition on cell proliferation in a panel of 26 human breast cancer cell lines. (A) Cells were transfected with either pooled cyclin D1 siRNA (siCyclin D1) or Non-targeting siRNA control at the indicated concentrations and BrdU incorporation measured 72 hours post-transfection. Western blot analyses show efficiency of siRNA-mediated inhibition of cyclin D1 expression in the representative human breast cancer cell lines. (B) Cells were treated with PD0332991 at the indicated concentrations for 48 hours followed by measurement of BrdU incorporation. Data are expressed as percentage of BrdU incorporation in cells treated with PD0332991 relative to cells treated with vehicle. ER+ cell lines are highlighted in green colour and ER-cell lines are highlighted in red colour.

Figure S4 The effects of CDK2 inhibitor SNS-032 on cell proliferation in a panel of 17 human breast cancer cell lines. Cells were treated with SNS-032 at various concentrations for 48 hours followed by measurement of BrdU incorporation. The IC_{50} value for each cell line was determined.

Figure S5 The effects of CDK1 inhibition on cell cycle progression and cell apoptosis in human breast cancer cells. (A) Cells were transfected with either pooled CDK1 siRNA (siCDK1) or Non-targeting siRNA control (siCON) at 10 nM and harvested 3 days post-transfection for cell cycle analysis by flow cytometry. Representative histograms are shown. (B) AU565, SKBR3 and MDA-MB-175 cells transfected with MYC siRNA (siMYC) or siCON were treated with either RO-3396 or CGP74514A at 5 μM for 48 hours. The whole cell lysates were harvested and subjected to Western blotting.

Table S1 Optimised transfection conditions in a 96-well plate format for human breast cancer cell lines.

Table S2 The sensitivity of human breast cancer cell lines to MYC siRNA.

Table S3 The sensitivity of human breast cancer cell lines to cyclin D1 siRNA, PD0332991 and SNS-032.

Table S4 Molecular features of human breast cancer cell lines.

Figure S1

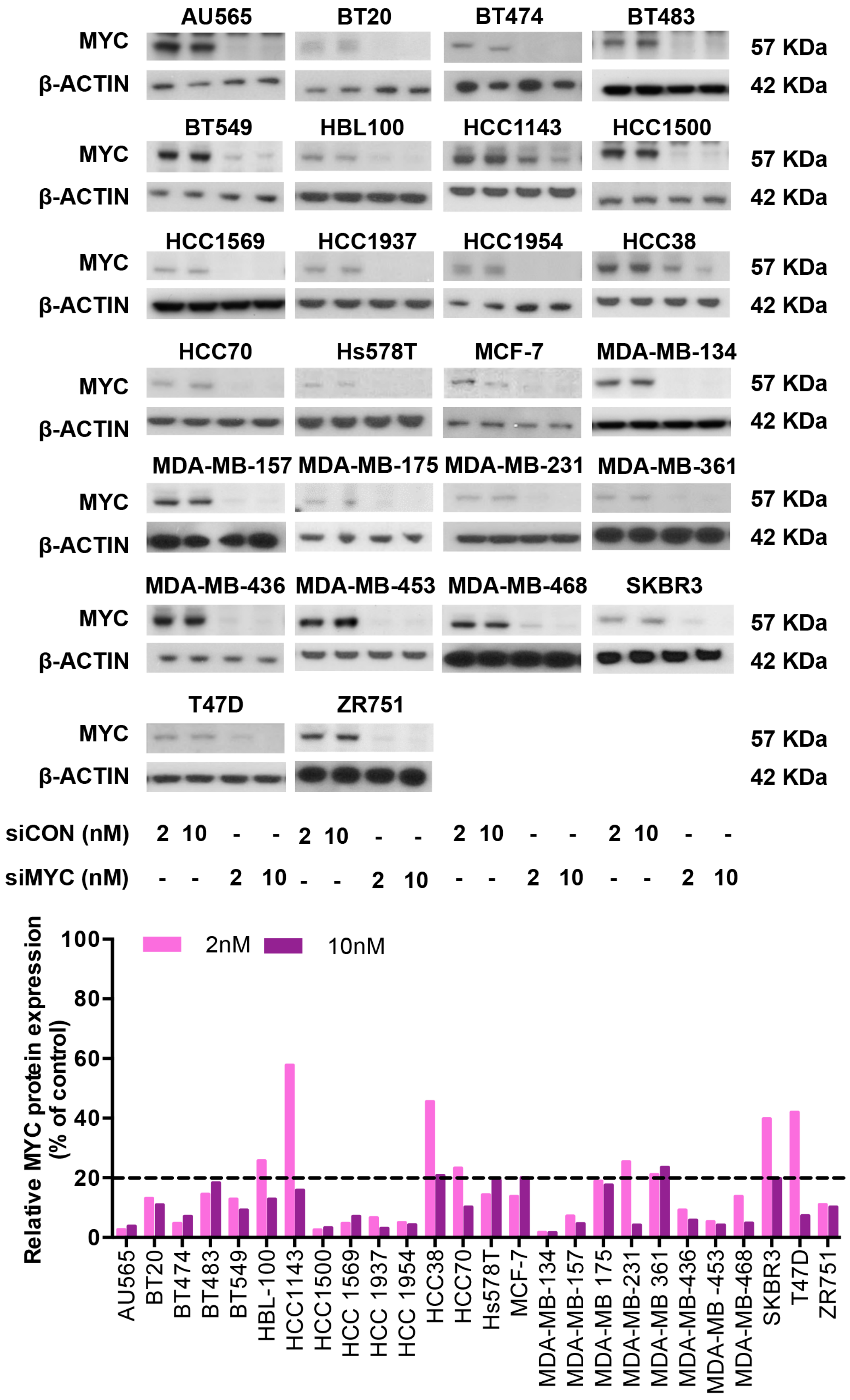
AU565
BT20
BT474
BT483
BT549
HBL100
HCC1143
HCC1500
HCC1569
HCC1937
HCC1954
HCC38
HCC70
Hs578T
MCF-7
MDA-MB-134
MDA-MB-157
MDA-MB-175
MDA-MB-231
MDA-MB-361
MDA-MB-436
MDA-MB-453
MDA-MB-468
SKBR3
T47D
ZR751
MYC
β-ACTIN
57 KDa
42 KDa
siCON (nM) 2 10 - - 2 10 - - 2 10 - - 2 10 - -
siMYC (nM) - - 2 10 - - 2 10 - - 2 10 - - 2 10
Relative MYC protein expression (% of control)
2nM
10nM
0
20
40
60
80
100
AU565
BT20
BT474
BT483
BT549
HBL-100
HCC1143
HCC1500
HCC 1569
HCC 1937
HCC 1954
HCC38
HCC70
Hs578T
MCF-7
MDA-MB-134
MDA-MB-157
MDA-MB 175
MDA-MB-231
MDA-MB 361
MDA-MB-436
MDA-MB -453
MDA-MB-468
SKBR3
T47D
ZR751

Figure S2

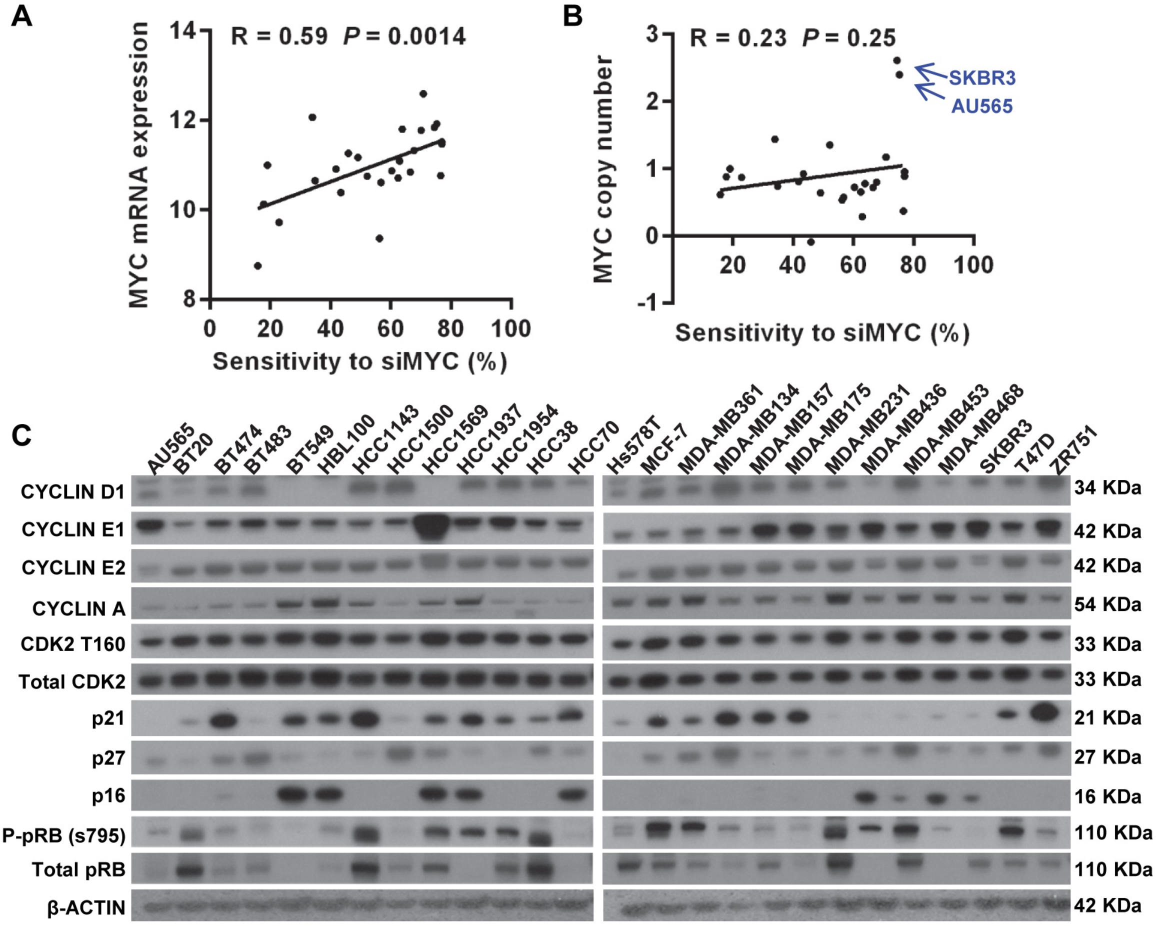
A
R = 0.59 P = 0.0014
MYC mRNA expression
Sensitivity to siMYC (%)
B
R = 0.23 P = 0.25
MYC copy number
SKBR3
AU565
Sensitivity to siMYC (%)
C
AU565
BT20
BT474
BT483
BT549
HBL100
HCC1143
HCC1500
HCC1569
HCC1937
HCC1954
HCC38
HCC70
Hs578T
MCF-7
MDA-MB361
MDA-MB134
MDA-MB157
MDA-MB175
MDA-MB231
MDA-MB436
MDA-MB453
MDA-MB468
SKBR3
T47D
ZR751
CYCLIN D1
CYCLIN E1
CYCLIN E2
CYCLIN A
CDK2 T160
Total CDK2
p21
p27
p16
P-pRB (s795)
Total pRB
β-ACTIN
34 KDa
42 KDa
42 KDa
54 KDa
33 KDa
33 KDa
21 KDa
27 KDa
16 KDa
110 KDa
110 KDa
42 KDa

Figure S3

A

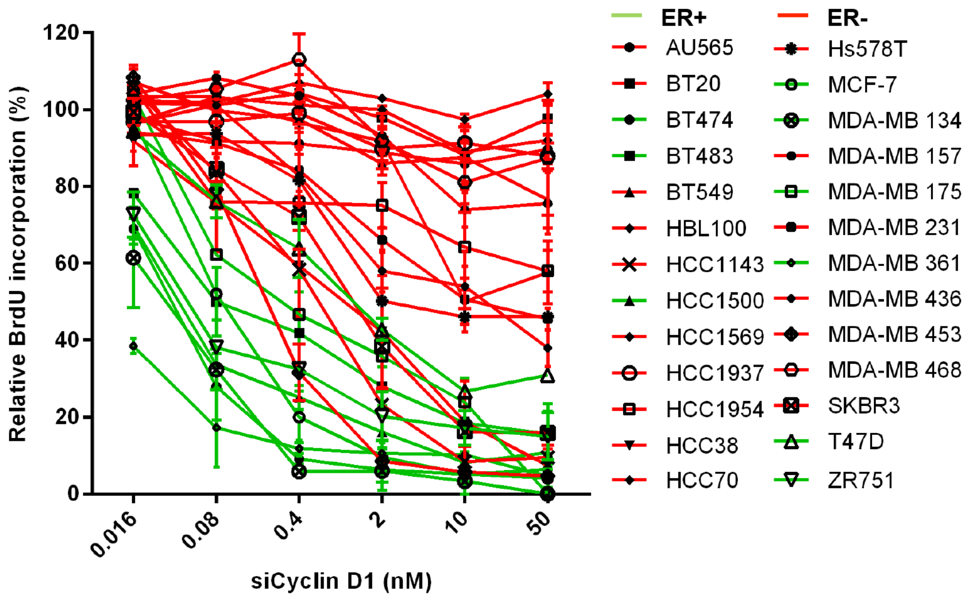
Relative BrdU incorporation (%)
0
20
40
60
80
100
120
0.016
0.08
0.4
2
10
50
siCyclin D1 (nM)
ER+
ER-
AU565
BT20
BT474
BT483
BT549
HBL100
HCC1143
HCC1500
HCC1569
HCC1937
HCC1954
HCC38
HCC70
Hs578T
MCF-7
MDA-MB 134
MDA-MB 157
MDA-MB 175
MDA-MB 231
MDA-MB 361
MDA-MB 436
MDA-MB 453
MDA-MB 468
SKBR3
T47D
ZR751

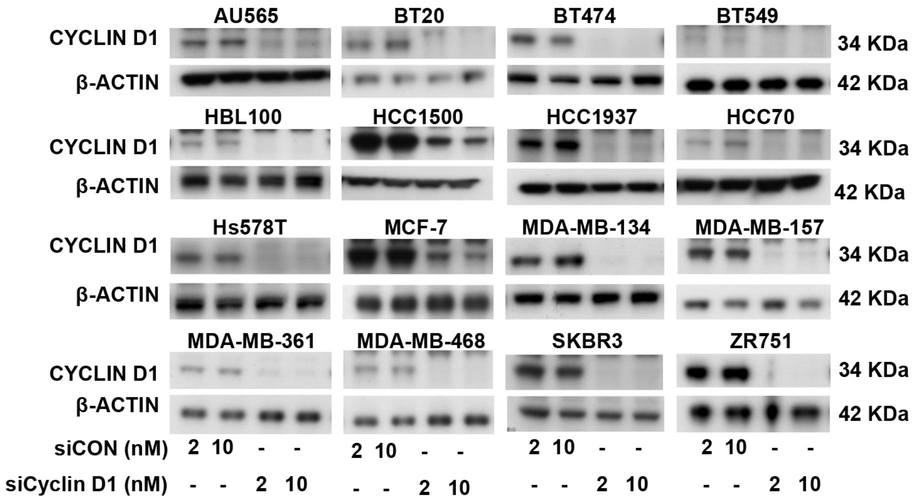
AU565
BT20
BT474
BT549
HBL100
HCC1500
HCC1937
HCC70
Hs578T
MCF-7
MDA-MB-134
MDA-MB-157
MDA-MB-361
MDA-MB-468
SKBR3
ZR751
CYCLIN D1
β-ACTIN
34 KDa
42 KDa
siCON (nM) 2 10 - -
siCyclin D1 (nM) - - 2 10

B

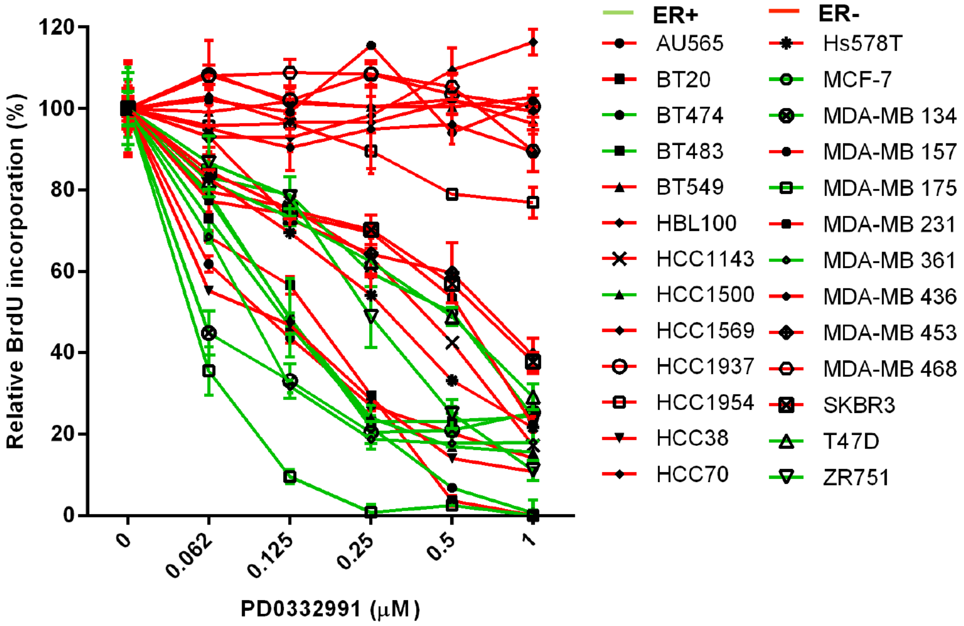
Relative BrdU incorporation (%)
0
20
40
60
80
100
120
0
0.062
0.125
0.25
0.5
1
PD0332991 (μM)
ER+
ER-
AU565
BT20
BT474
BT483
BT549
HBL100
HCC1143
HCC1500
HCC1569
HCC1937
HCC1954
HCC38
HCC70
Hs578T
MCF-7
MDA-MB 134
MDA-MB 157
MDA-MB 175
MDA-MB 231
MDA-MB 361
MDA-MB 436
MDA-MB 453
MDA-MB 468
SKBR3
T47D
ZR751

Figure S4

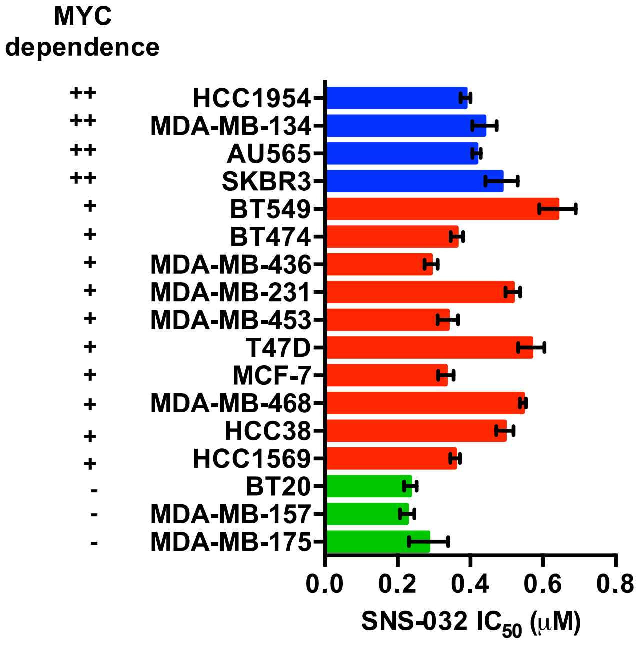
MYC
dependence
++
++
++
++
+
+
+
+
+
+
+
+
+
+
-
-
-
HCC1954
MDA-MB-134
AU565
SKBR3
BT549
BT474
MDA-MB-436
MDA-MB-231
MDA-MB-453
T47D
MCF-7
MDA-MB-468
HCC38
HCC1569
BT20
MDA-MB-157
MDA-MB-175
0.0
0.2
0.4
0.6
0.8
SNS-032 IC50 (μM)

Figure S5

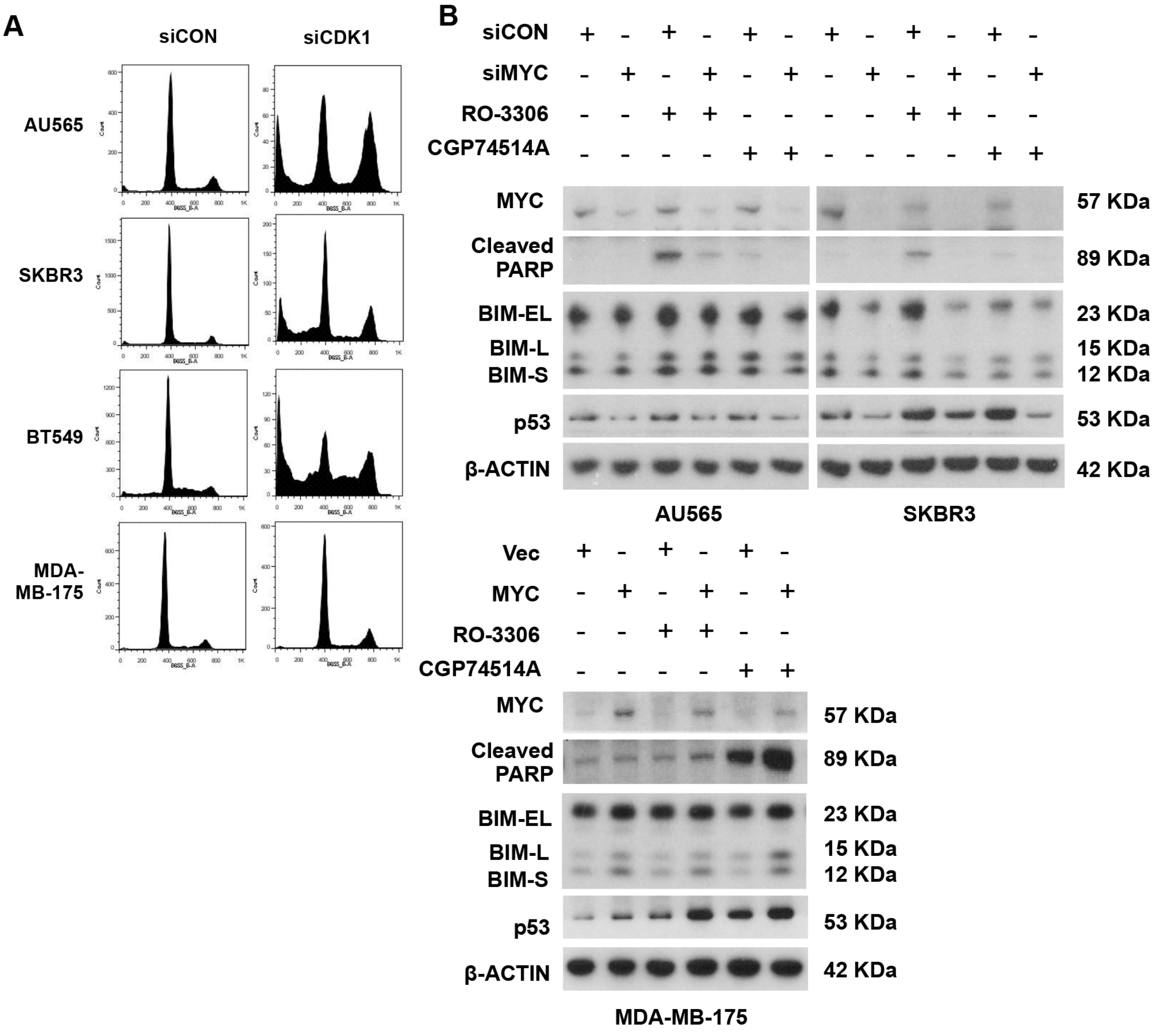
A
siCON
siCDK1
AU565
SKBR3
BT549
MDA-MB-175
B
siCON
siMYC
RO-3306
CGP74514A
MYC
Cleaved PARP
BIM-EL
BIM-L
BIM-S
p53
β-ACTIN
57 KDa
89 KDa
23 KDa
15 KDa
12 KDa
53 KDa
42 KDa
AU565
SKBR3
Vec
MYC
RO-3306
CGP74514A
MDA-MB-175

Table S1 Optimised transfection conditions in a 96-well plate format for human breast cancer cell lines.

**Cell line**	**Transfection reagent**	**Lipid volume per well (µL)**	**Cell density per well**
AU565	DharmaFECT 3	0.25	4000
BT20	DharmaFECT 2	0.12	4000
BT474	DharmaFECT 1	0.12	4000
BT483	DharmaFECT 1	0.25	4000
BT549	DharmaFECT 1	0.12	4000
HBL-100	DharmaFECT 2	0.25	4000
Hs578T	DharmaFECT 3	0.25	3000
HCC1143	Lipofectamine 2000	0.25	5000
HCC1187	DharmaFECT 3	0.12	4000
HCC1500	Lipofectamine 2000	0.25	6000
HCC1569	Lipofectamine 2000	0.25	4000
HCC1937	Lipofectamine 2000	0.12	4000
HCC1954	Lipofectamine 2000	0.25	5000
HCC38	DharmaFECT 2	0.12	4000
HCC70	Lipofectamine 2000	0.25	5000
MCF-7	DharmaFECT 1	0.25	4000
MDA-MB-134	DharmaFECT 3	0.25	4000
MDA-MB-157	DharmaFECT 2	0.25	6000
MDA-MB-175	Lipofectamine 2000	0.5	6000
MDA-MB-231	DharmaFECT 1	0.12	2000
MDA-MB-361	DharmaFECT 3	0.25	4000
MDA-MB-436	DharmaFECT 2	0.25	4000
MDA-MB-453	DharmaFECT 3	0.25	4000
MDA-MB-468	DharmaFECT 1	0.25	4000
SKBR3	DharmaFECT 2	0.25	4000
T47D	DharmaFECT 1	0.12	4000
ZR751	DharmaFECT 2	0.25	4000

Table S2 The sensitivity of human breast cancer cell lines to MYC siRNA.

Cell line	MYC Dependence	Inhibition of BrdU incorporation by MYC siRNA (%)		Relative MYC protein expression	Relative phospho-MYC expression
		Mean	SEM		
AU565	++	75.32	5.52	2.90	0.96
BT20	-	19.09	10.39	0.70	0.20
BT474	+	66.52	10.55	1.58	0.49
BT483	+	43.44	0.81	0.94	0.20
BT549	+	67.70	0.22	2.22	0.81
HBL100	+	49.61	4.17	1.59	0.60
HCC1143	+	70.78	6.46	1.93	0.43
HCC1500	-	22.95	3.58	1.75	0.19
HCC1569	+	33.96	5.43	1.76	0.56
HCC1937	+	41.88	14.55	1.46	0.43
HCC1954	++	76.92	2.13	1.88	0.36
HCC38	+	34.89	5.05	1.09	0.22
HCC70	+	60.36	12.23	1.86	0.93
Hs578T	++	76.98	8.80	1.08	0.72
MCF-7	+	52.18	11.97	1.00	0.20
MDA-MB-134	++	76.54	1.26	1.48	0.50
MDA-MB-157	-	17.82	4.68	0.41	0.18
MDA-MB-175	-	12.69	12.78	0.17	0.27
MDA-MB-231	+	62.95	6.95	1.77	1.00
MDA-MB-361	+	56.28	4.39	0.78	0.27
MDA-MB-436	+	63.82	9.08	1.44	0.58
MDA-MB-453	+	62.48	3.23	1.12	0.53
MDA-MB-468	+	45.94	7.77	1.10	0.80
SKBR3	++	74.50	1.41	1.39	0.52
T47D	+	56.75	1.41	1.04	0.60
ZR751	+	49.06	9.94	0.82	0.21

Table S3 The sensitivity of human breast cancer cell lines to cyclin D1 siRNA, PD0332991 and SNS-032.

Cell line	Inhibition of BrdU incorporation (%) 2 nM cyclin D1 siRNA		Inhibition of BrdU incorporation (%) 0.25 µM PD0332991		SNS-032 IC_{50} (µM)	
	**Mean**	**SEM**	**Mean**	**SEM**	**Mean**	**SEM**
AU565	11.21	3.08	73.04	1.18	0.42	0.01
BT20	2.09	2.98	70.46	0.86	0.24	0.02
BT474	93.65	5.30	78.32	5.37	0.36	0.02
BT483	72.03	5.01	76.92	1.50		
BT549	13.97	3.04	0.00	2.39	0.64	0.05
HBL100	8.15	3.78	1.74	9.89		
HCC1143	76.79	4.25	36.62	2.22		
HCC1500	83.84	0.85	76.02	1.01		
HCC1569	0.00	1.10	0.00	0.68	0.36	0.01
HCC1937	10.21	3.01	0.00	2.68		
HCC1954	24.94	5.87	10.38	4.49	0.39	0.01
HCC38	57.76	14.54	71.53	1.53	0.50	0.02
HCC70	0.00	1.02	3.47	0.90		
Hs578T	49.75	3.32	45.83	4.43		
MCF-7	90.43	2.55	40.46	3.12	0.33	0.02
MDA-MB-134	94.00	2.92	79.62	2.72	0.44	0.03
MDA-MB-157	6.78	4.61	0.00	2.49	0.23	0.02
MDA-MB-175	64.22	4.47	99.27	1.86	0.29	0.05
MDA-MB-231	33.88	3.21	30.42	1.50	0.52	0.02
MDA-MB-361	89.39	2.75	81.25	0.92		
MDA-MB-436	47.1	5.44	5.06	10.96	0.29	0.02
MDA-MB-453	91.48	0.01	35.74	4.97	0.34	0.03
MDA-MB-468	8.17	7.20	0.00	3.29	0.55	0.01
SKBR3	31.28	1.56	29.78	3.59	0.49	0.04
T47D	57.23	2.89	37.68	1.54	0.57	0.04
ZR751	79.79	6.33	51.24	7.49		

Table S4 Molecular features of human breast cancer cell lines.

Cell line	MYC Dependence[#]	Subtype*	ER status*	PR status*	HER2 amplified*	pRb status[¶]
AU565	++	Luminal	-	-	+	+
BT20	-	Basal A	-	-	-	+
BT474	+	Luminal	+	+	+	+
BT483	+	Luminal	+	+	-	+
BT549	+	Basal B	-	-	-	-
HBL100	+	Basal B	-	-	-	+
HCC1143	+	Basal A	-	-	-	+
HCC1500	-	Luminal	+	+	-	+
HCC1569	+	Basal A	-	-	+	+
HCC1937	+	Basal A	-	-	-	-
HCC1954	++	Basal A	-	-	+	+
HCC38	+	Basal B	-	-	-	+
HCC70	+	Basal A	-	-	-	-
Hs578T	++	Basal B	-	-	-	+
MCF-7	+	Luminal	+	+	-	+
MDA-MB-134	++	Luminal	+	-	-	+
MDA-MB-157	-	Basal B	-	-	-	+
MDA-MB-175	-	Luminal	+	-	-	+
MDA-MB-231	+	Basal B	-	-	-	+
MDA-MB-361	+	Luminal	+	+	+	+
MDA-MB-436	+	Basal B	-	-	-	-
MDA-MB-453	+	Luminal	-	-	+	+
MDA-MB-468	+	Basal A	-	-	-	-
SKBR3	++	Luminal	-	-	+	+
T47D	+	Luminal	+	+	-	+
ZR751	+	Luminal	+	-	-	+

Determined from the present study. Based on the sensitivity to 10 nM MYC siRNA, breast cancer cells were classified into MYC-highly dependent (++), dependent (+), and MYC-independent (-) cells.

[*] References from the studies by Neve *et al.* [1], and Kao *et al.* [2].

[¶] Determined from the present study.

1. Neve RM, Chin K, Fridlyand J, Yeh J, Baehner FL, Fevr T, Clark L, Bayani N, Coppe JP, Tong F, Speed T, Spellman PT, DeVries S, Lapuk A, Wang NJ, Kuo WL, Stilwell

JL, Pinkel D, Albertson DG, Waldman FM, McCormick F, Dickson RB, Johnson MD, Lippman M, Ethier S, Gazdar A, Gray JW: **A collection of breast cancer cell lines for the study of functionally distinct cancer subtypes**. *Cancer Cell* 2006, **10**(6):515-527.
2. Kao J, Salari K, Bocanegra M, Choi YL, Girard L, Gandhi J, Kwei KA, Hernandez-Boussard T, Wang P, Gazdar AF, Minna JD, Pollack JR: **Molecular profiling of breast cancer cell lines defines relevant tumor models and provides a resource for cancer gene discovery**. *PLoS One* 2009, **4**(7):e6146.
